# How do the two visual streams interact with each other?

**DOI:** 10.1007/s00221-017-4917-4

**Published:** 2017-03-02

**Authors:** A. D. Milner

**Affiliations:** 1 Durham University, Durham, UK; 2 Department of Psychology, Science Laboratories, Durham University, South Road, Durham, DH1 3LE UK

**Keywords:** Ventral, Dorsal, Streams, Visual, Cortex

## Abstract

The current consensus divides primate cortical visual processing into two broad networks or “streams” composed of highly interconnected areas (Milner and Goodale 2006, 2008; Goodale 2014). The ventral stream, passing from primary visual cortex (V1) through to inferior parts of the temporal lobe, is considered to mediate the transformation of the contents of the visual signal into the mental furniture that guides memory, recognition and conscious perception. In contrast the dorsal stream, passing from V1 through to various areas in the posterior parietal lobe, is generally considered to mediate the visual guidance of action, primarily in real time. The brain, however, does not work through mutually insulated subsystems, and indeed there are well-documented interconnections between the two streams. Evidence for contributions from ventral stream systems to the dorsal stream comes from human neuropsychological and neuroimaging research, and indicates a crucial role in mediating complex and flexible visuomotor skills. Complementary evidence points to a role for posterior dorsal-stream visual analysis in certain aspects of 3-D perceptual function in the ventral stream. A series of studies of a patient with visual form agnosia has been instrumental in shaping our knowledge of what each stream can achieve in isolation; but it has also helped us to tease apart the relative dependence of parietal visuomotor systems on direct bottom-up visual inputs versus inputs redirected via perceptual systems within the ventral stream.

## Introduction

Otto Creutzfeldt, one of the pre-eminent European neuroscientists of the post-war period, was a major player in the birth and development of *Experimental Brain Research*, and indeed succeeded Sir John Eccles as the chief editor from 1976, his stewardship ending only with his premature death in January 1992. In the 1980s, Creutzfeldt published two papers (1981, 1985), in which he recognized the crucial importance of output connections for a full understanding of the functional roles of different cortical visual areas. It is of course a truism that all visual processing systems ultimately serve to guide behaviour; otherwise they would never have evolved. Creutzfeldt, however, pointed out that quite different patterns of neural efferents had been observed in the then-known cortical visual areas, and presciently proposed that “not only different features of a stimulus are represented but also different behavioural responses to stimuli” (Creutzfeldt 1985). This insight has been amply confirmed in a broad array of subsequent research in visual neuroscience (summarized by Milner and Goodale 2006). In particular, we know that there are direct pathways from the occipito-parietal “dorsal stream” to subcortical structures such as the superior colliculus, and to other brainstem structures that control the eye muscles and parts of the spinal cord that control the limbs (Glickstein et al. 1985; Baizer et al. 1993; Borra et al. 2014). Areas in the occipito-temporal ventral stream have few or none of these direct connections with motor systems. Instead, the ventral stream interfaces with structures in the temporal and frontal lobes that have been implicated in memory, emotion, and social behaviour, including the amygdala and other mesial temporal structures (Iwai and Yukie 1987; Baizer et al. 1993). The Two Visual Systems model developed by Milner and Goodale (1995, 2006; Goodale and Milner 1992; Jeannerod and Rossetti 1993) builds upon Creutzfeldt’s insight, conceiving “the functional role of the two streams as largely defined in terms of their outputs to other regions of the brain, what we might call the ‘consumers’ of those outputs, and the tasks those consumers serve” (Foley et al. 2016).

The existence of these two interconnected clusters of visual areas in the nonhuman primate neocortex is amply documented in anatomical studies, as collated pictorially by Felleman and Van Essen (1991) and mathematically by Young (1992). There may be some value in regarding the dorsal stream itself as being split into two interacting parts (Rizzolatti and Matelli 2003), and perhaps likewise the ventral stream (Aflalo and Graziano 2011). What is not in dispute, however, is that several of the early connectional studies showed—and new anatomical studies continue to show—clear, if less profuse, interconnections between the two visual streams themselves. For example, investigations have found bidirectional projections between temporal area TEO and the lateral intraparietal area, LIP (Distler et al. 1993; Webster et al. 1994). Projections to inferior temporal cortex (TE) in monkeys have been reported more recently from areas within the intraparietal sulcus including the anterior intraparietal area, AIP (Zhong and Rockland 2003; Borra et al. 2008, 2010).

Clearly then, although anatomy tells us that visual areas may work within cooperative conglomerates to perform distinctive roles, these conglomerates also need to talk to each other. The most obvious role for this inter-stream communication would be to provide an integration between the two disparate systems, such that the animal is provided with a unitary visual life. The likelihood of such a role was recognized in the earliest formulations of Two Visual Systems theory (Goodale and Milner 1992; Milner and Goodale 1993, 1995). For example, in the final section of the Milner and Goodale (1995) book, we wrote:

“efficiently programmed and coordinated behaviour requires that neither the ventral nor the dorsal stream should work in isolation: they should cooperate. It is, therefore, to be expected that there will be reciprocal cross-connections between areas in the two streams, and there is extensive anatomical evidence that this is so (Felleman and Van Essen 1991).

Understanding these interactions would take us some way towards answering what is one of the central questions in modern neuroscience: how is sensory information transformed into purposeful acts?”

Cloutman (2013) outlines three potential forms of cross-stream interaction:

1. computations along the two pathways proceed strictly independently and in parallel, reintegrating at some ‘terminal’ stage of processing within a shared target brain region (the ‘independent processing’ account);
2. processing along the separate pathways is modulated by the existence of feedback loops which transmit information from ‘downstream’ brain regions, including information processed along the complementary stream (the ‘feedback’ account); and
3. information is transferred between the two systems at multiple stages and locations along their processing pathways (the ‘continuous cross-talk’ account).

In the present article I will concentrate on possibility (3), though a resolution of the problems of visual integration and mental unification alluded to in the above quotation from Milner and Goodale (1995) is likely to involve Cloutman’s possibilities (1) and (2) (see Goodale and Milner 2004; Milner and Goodale 2006). The former may well operate via common projections to the lateral prefrontal cortex or superior temporal sulcus (Borra et al. 2008, 2010); the latter via back-projections to early retinotopic cortical areas (Rockland and Van Hoesen 1994; Borra and Rockland 2011).

## Visuomotor performance in patient D.F.

A major inspiration for Milner and Goodale’s original formulation of the Two Visual Systems model was provided by a series of behavioural observations carried out with a patient (D.F.) suffering from visual form agnosia. These studies showed that D.F. was able to perform manual actions guided by visual information that was not available to her for making perceptual reports. First, Milner et al. (1991) used a vertically mounted disc containing a large slot which was randomly set at different angles. They found that D.F.’s attempts to describe or otherwise report the orientation of the slot showed little or no relationship to its orientation. When asked to insert her hand or a hand-held card into the slot from a starting position an arm’s length away, however, she showed no difficulty. Video recordings showed that her hand began to rotate in the appropriate direction as soon as it left the start position. In short, although she could not report the orientation of the slot, she could direct her hand or a card into it without difficulty. The results were replicated by Goodale et al. (1991), who showed that D.F. was quite unable to match the orientation of a slot using a hand-held plaque while keeping her arm stationary, though she performed accurately when ‘posting’ the same plaque into the slot. A similar dissociation was later found with solid rectangular blocks when presented in front of her at different orientations: D.F.’s perceptual judgements of the stimuli were poor, yet her grasping movements to pick them up were accurate, and were preceded by normal anticipatory orientation of the wrist during the course of the reaching movement (Carey et al. 1996).

Goodale et al. (1991) observed similar dissociations between perceptual report and visuomotor control in D.F. when she had to deal with the intrinsic properties of objects such as their width and shape. Thus, D.F.’s hand exhibited normal anticipatory shaping as she reached out to pick up blocks of different width—ones that she could not distinguish perceptually. In one such test, solid blocks of matched surface area but different widths (based on a shape discrimination task devised by Efron 1969) were used. Healthy subjects adjust their fingerthumb separation in advance of arrival at the object during such reaching behaviour (Jeannerod 1986; Jakobson and Goodale 1991), reaching a maximum grip aperture at about 75% of the way toward the object. This maximum aperture is strongly related to the width of the object. D.F. showed this visual scaling of her grip size quite normally during reaching. Yet when she was asked to use her finger and thumb to make a perceptual judgement of the object’s width on a separate series of trials (in a manner analogous to the matching task she had carried out earlier with the card and slot), her responses were unrelated to the stimulus, and showed high variation from trial to trial.

Finally, D.F.’s sensitivity to the outline shape of irregular flat objects was tested. To pick up the object successfully, the fingers and thumb had to be placed at appropriate opposition points on the object’s perimeter. D.F.’s performance with these stimuli yielded another clear dissociation between perception and action: she was able to position her index finger and thumb in stable positions on either side of each object during grasping, but was quite unable to discriminate one object from another (Goodale et al. 1994a). In contrast, the authors found that a patient with bilateral parietal damage and optic ataxia (R.V.) failed to place her fingers correctly on the objects, with the result that they would frequently slip out of her grasp. Yet R.V. could readily distinguish these objects from one another.

Taken together, these findings clearly indicate the preserved operation in D.F. of a system for visual control of manual actions on the basis of orientation, width, and shape, despite her profound visual form agnosia. Indeed in the study mentioned above by Carey et al. (1996), we further confirmed that orientation and width could work together in concert to guide D.F.’s hand and fingers, in that she was able to reach out and grasp solid plaques of different dimensions placed at varying orientations in front of her, with an accuracy equal to healthy controls. Following the early studies of D.F.’s performance, it was proposed that her lesion had critically damaged the ventral stream of cortical processing, but left the bulk of the dorsal stream intact (Goodale and Milner 1992; Milner and Goodale 1993, 1995). That is, we proposed that her spared visuomotor capacities reflected a relatively intact dorsal stream, in the presence of a severely compromised ventral stream. Recent functional structural MRI studies of D.F. indicate that actually there is some bilateral (particularly right-hemisphere) posterior parietal damage present in her brain (James et al. 2003; Bridge et al. 2013). Yet the visuomotor area most concerned with grasping (AIP) is robustly and selectively activated during prehension in D.F., whereas her ventral stream area LOC (lateral occipital cortex) appears to be completely destroyed bilaterally, and no selective activation to visual shapes is detectable (James et al. 2003).

Given this pattern of brain damage, it is clear that the information that area LOC would normally share with the dorsal stream would not be available in D.F.: in other words, not only would she suffer the direct effects of bilateral LOC damage on shape perception, she would also suffer the indirect effects of losing ventral-stream influence on dorsal-stream processing. This has caused a number of specific problems in visuomotor tasks whose complexity exceeds the basic ones that are described above. Some of the ventral-to-dorsal stream interactions whose absence underlie these impairments were predicted on the basis of the model as first set out by Goodale and Milner (1992), for example in delayed action (Goodale et al. 1994c); and others were predicted when the ramifications of the model were further thought out, such as in visual judgements of weight (Dijkerman et al. 2004). Yet other ventral-dorsal interactions, however, were not predicted, but became apparent as we sought to refine our understanding through empirical investigation, such as in D.F.’s responses to multiple visual orientations (Goodale et al. 1994b); and also unpredicted were the dorsal-to-ventral interactions that are reviewed later in this paper, such as those involved in stereoscopic depth perception.

## Evidence for ventral-to-dorsal traffic

### Multiple orientations

Although D.F. could use the orientation of a target to control the orientation of her hand in a posting or grasping task, the question arose as to whether she could use the orientation of a more complex target stimulus to control hand rotation during posting or grasping. We first explored this question by asking her to post a T-shaped object into a T-shaped aperture (Goodale et al. 1994b). On different test trials, the target aperture was presented at different orientations, such that its principal axis was oriented at ±30° or ±60° from the vertical. We found that D.F. succeeded in smoothly inserting the T shape on about half of the trials; but on the other trials her errors were almost always made at approximately 90°. This result confirms that D.F. is able to use the orientation of one visible edge to determine her manual posting behaviour, but suggests that she cannot combine two visual orientations to form a composite shape to guide such actions. In a second study designed to test D.F.’s spared visuomotor abilities with multiple orientations, we found that her hand orientation *en route* towards grasping a cross-shaped object was insensitive to changes in orientation of the object, averaging the same default wrist posture whatever the stimulus orientation (Carey et al. 1996).

The behavioural variable being measured in these experiments was the orientation of the wrist, either directly (in cross grasping) or indirectly by virtue of turning the T-shaped object to ‘post’ it. Clearly this element of prehension has only one degree of freedom, and in the absence of ventral stream processing may be driven not by shape as such, but rather by a single dominant axis present in the display. Such a form of primitive visuomotor control would perhaps account for the results of the T-posting study. A clear principal axis, however, was not present in the cross experiment as the stimulus was doubly symmetrical: as a result it may have been incapable of controlling wrist orientation at all without the additional influence of ventral-stream shape processing. It may be hypothesized that in D.F.’s isolated dorsal stream, rotation of the wrist is only sensitive to one major visual axis at a time, rendering it limited to translating visual orientation into oriented action reliably only with stimuli where there is a single major axis. Recent psychophysical evidence supports this idea (Almeida et al. 2014). If this is so, then a healthy person’s performance of these two more visually complex tasks may depend upon input from shape processing systems in the ventral stream that are able to upgrade such a first-order orientation visuomotor channel in the dorsal stream into a more flexible one that can simultaneously handle multiple visual axes.

### Delayed action

D.F.’s ability to scale her grasp to the size of a goal object is striking, but nevertheless has certain revealing limitations. In a seminal early study, Goodale et al. (1994c) examined the effects of interposing a delay between briefly presenting an object to D.F. and then allowing her to reach out to perform a grasp as if the object were still there. In control subjects, grip size still correlated well with object width, even for delays as long as 30 s. In D.F., however, all evidence of grip scaling had disappeared after a delay of only 2 s. Kinematic analysis showed that even the grasping movements of healthy subjects in the delay condition took a very different form from those directed at objects that were physically present. It was inferred that when making such ‘pantomimed’ grasps, healthy subjects had to use a stored perceptual representation of the object generated in the ventral stream to supplement the direct dorsal-stream route dedicated to normal target-directed grasping. In other words, delayed action would require some form of cross-stream information transmission in the ventral-to-dorsal direction at the time of the action. This interpretation was borne out by the later discovery that transcranial magnetic stimulation (TMS applied to the dorsal stream (area AIP) in healthy subjects compromised both immediate and delayed grasping, whereas TMS to the ventral stream (area LOC) compromised only delayed grasping (Cohen et al. 2009). Clearly D.F’s brain damage would have precluded the use of this circuitous route to the dorsal steam via LOC. In a later study it was found using fMRI that both LOC and also early visual cortex (including V1) were re-activated at the end of the delay period—even though the participants remained in complete darkness with no visual stimulation at the time of the action (Singhal et al. 2013). (As an aside, the authors observed higher activation for grasping than reaching within early visual cortex, during both vision and subsequent action execution. This may indicate the existence of downstream priming that could affect both streams in the manner of Cloutman’s (2013) second putative cross-stream mechanism.)

D.F. performs accurately in reaching towards individual items distributed within her visual field, despite a severe deficit in perceiving spatial relationships among the items (Carey et al. 2006). Milner et al. (1999), however, found that here too the imposition of a delay impaired D.F.’s performance. Using laterally located targets (four LEDs spaced 2.5 deg apart on either side of a fixation point), they reported that D.F.’s errors were similar to those of healthy controls when she was allowed to respond immediately, but were 3 times greater than control values after a 10 s delay. Similar results have been reported recently in a patient with hemiagnosia caused by unilateral ventral-stream damage (Cornelsen et al. 2016). This patient performed considerably worse than controls for the most peripheral contralesional target during delayed reaching, but was proficient at immediate reaching. D.F. also showed a comparable dissociation when she was asked to make saccadic eye movements to a target location, either directly or after a delay when the target was no longer there (Milner et al. 1999; Rossit et al. 2010). In the latter case her accuracy dropped precipitously.

In summary, the behaviour of D.F. and of healthy subjects in delayed grasping and reaching is consistent with an assumption that visuomotor mechanisms within the dorsal stream, if left to their own devices, operate very much in the ‘here and now’. When movements have to be generated after even short delays, the brain has to make use of stored perceptual representations constructed within the ventral-stream via cross-stream inputs. Unfortunately of course this ventral-stream source of information is no longer available for D.F.’s dorsal stream to receive.

### Grasping spatial relationships

Dijkerman et al. (1998) tested D.F. on a complex prehension task in which she was presented with transparent circular discs, each of which had circular holes cut in it. D.F. was asked to reach out and grasp the disc by placing her fingers through the holes. The discs either had three holes (for forefinger, middle finger, and thumb) or two holes (for forefinger and thumb). In the three-hole task, D.F. was quite unable to adjust her grip aperture with respect to the distance between the forefinger and thumb holes or her hand orientation with regard to their relative orientation of the holes. Although she *was* able to orient her hand appropriately for the two-hole disks, she still remained unable to adjust her grip aperture to the distance between the holes. McIntosh et al. (2004) subsequently clarified these findings. First, they replicated the earlier findings that D.F. was unable to produce normal prehension movements when attempting to grasp transparent stimuli by placing her digits into holes. However, they went on to show, using parallel pairs of elongated stimuli, that D.F. was perfectly able to scale her grip with respect to the separation between a pair of objects, just as well as with respect to the width of a single stimulus.

These findings are consistent with the proposal that allocentric processing of spatial information where three or more locations need to be combined requires access to a functioning ventral stream, whether the information is being used to guide a motor response (Dijkerman et al. 1998; McIntosh et al. 2004) or not (Murphy et al. 1998; Carey et al. 2006, 2009). If this is correct, then clearly grasping a 3-hole disc would require ventral-to-dorsal crosstalk. In addition, there seems to be a separate problem when the task requires insertion of digits into particular holes in an object rather than the more natural grasping of outer surfaces of objects. Just as concluded above for multiple contour orientations, although simple objects may offer themselves directly to the dorsal stream for grasping, an intact ventral stream seems to be required to respond appropriately to complex stimuli. This limitation on the capacity of dorsal visuomotor channels again most probably demands the intercession of ventral-to-dorsal crosstalk.

### Grasping orientation in depth

Dijkerman et al. (1996) devised a grasping task designed to investigate D.F.’s ability to use binocular and monocular information about the orientation of an object in the depth plane for perceptual and visuomotor purposes. A square plaque was presented at 7 different slants for subjects to reach out and grasp using a precision grip, under binocular and monocular viewing conditions. (In separate testing they were asked to match the slant of the target using a hand-held plaque: we will return to this later). D.F.’s scaling of her handgrip orientation was found to be normal under binocular conditions, but substantially impaired using monocular vision. This finding is consistent with reports that many neurons in the monkey’s intraparietal area CIP respond selectively to orientation in depth, and that many of these cells require binocular viewing of the target, becoming less responsive when one eye is occluded (Sakata et al. 1995; Shikata et al. 1996). Presumably when binocular vision is unavailable, the extraction of depth information for visuomotor control has to rely on pictorial cues like texture, illumination gradients, and (particularly relevant here) perspective. Other evidence indicates that perspective cues are not available to D.F. due to her ventral stream damage (Marotta et al. 1997; Mon-Williams et al. 2001), so that when deprived of binocular cues in Dijkerman et al.’s (1996) study, her performance inevitably deteriorated. Corroborative evidence was later obtained in another patient with visual form agnosia, S.B. (Lê et al. 2002), who showed similar results (Dijkerman et al. 2004). Taken together, this evidence strongly suggests that parietal visuomotor systems—unless informed by ventral stream crosstalk—are critically dependent on binocular input for processing orientation in depth.

Pursuing this logic further, Verhagen et al. (2008) argued that although both viewing conditions in Dijkerman et al.’s (1996) task are likely to engage both streams in healthy brains, the dorsal stream would need to rely more on inputs from the ventral stream as the relevance of pictorial depth cues increases. In particular, increasing the object slant increases the importance of pictorial cues like perspective whereas the presence of binocular cues decreases that need (Knill and Saunders 2003). Verhagen et al. (2008) used functional MRI to test for such dynamic cross-stream interactions and found that area AIP (in conjunction with ventral premotor cortex, PMv) and area LOC in the ventral visual stream showed differential slant-related responses, with activity increasing when monocular viewing conditions and increasing slant required the processing of pictorial depth cues. These conditions also increased the functional coupling of AIP with both LOC and PMv. They, therefore, argue that the trial-to-trial demands of the task modulate the extent to which the dorsal stream imports perceptual information into the prehension plan in an online fashion.

### Control of grip force

While the data from D.F. indicate that ventral-stream information about width and shape is not required for the dorsal stream to mediate accurate scaling of finger-thumb grip size with simple objects, there have long been suggestions that an equally important aspect of grip control may not be so autonomous. The grip forces we exert when picking up an object are normally tailored to its expected weight, which other things being equal, will vary with its apparent size (see Johansson and Cole 1992). But such expectations depend on learned associations, rather than being directly specified in the visual information available as we look at an object, and therefore would be expected a priori to rely on the kinds of visual processing for which the ventral stream is specialized (Milner and Goodale 2006). Data consistent with this interpretation come from studies using pictorial illusions of size, which have been shown to have little or no effect on the scaling of grip aperture in flight (Aglioti et al. 1995; Ganel et al. 2008). Such illusions nevertheless show a strong effect not only on size perception but also on the calibration of grip forces used to pick up a target object (Brenner and Smeets 1996; Jackson and Shaw 2000). That the ventral stream plays an important role in judging weight also gains support from preliminary evidence that both of our patients with visual form agnosia and ventral-stream lesions, D.F. and S.B., fail to show a significant visual size-weight illusion, which in some sense also depends on visually generated expectations about weight (McIntosh 2000; Dijkerman et al. 2004; but see also; Flanagan and Beltzner 2000). Yet in a separate test they both experienced a strong kinaesthetic size-weight illusion, in which they simply felt the size and shape of the objects while blindfolded.

More direct evidence of the role of the ventral stream in weight perception has recently been reported using functional MRI: using multivoxel pattern analysis, Gallivan et al. (2014) have found that the weight of an object being lifted is represented in specific “visual” areas in occipito-temporal cortex. Even more interestingly, the pattern of response in ventral stream visual areas varied according to whether an object’s predicted weight was based on repeated experience of lifting a specific object, or from associations between the surface properties (colour and texture) of the object and its weight. In the former case, the activations were biased towards lateral occipital cortex (associated with shape perception), while in the latter they were biased towards posterior fusiform areas close to the anterior part of the collateral sulcus (associated with surface properties: Cavina-Pratesi et al. 2010a, b). These results provide evidence that the ventral visual pathway is actively and flexibly engaged in processing object weight. Since it is known from TMS studies (Davare et al. 2007) that dorsal-stream area AIP is critically involved in grip-force control as well as in grip-size scaling, we may assume that there is constant direct and flexible traffic between this area and the ventral-stream areas representing shape and surface properties. That is, just as we saw in the previous section from Verhagen et al.’s (2008) work on the visuomotor control of grasping objects oriented in depth, we see here further evidence for inter-stream interactions being recruited dynamically according to the current behavioural demands.

### Semantic influences on action

Advance knowledge of object function allows us to fine-tune our actions to suit the objects we may interact with in our daily life. For example, D.F. makes mistakes in picking up everyday objects like tools and cutlery, not in mis-scaling her grip or mis-orienting her hand, but in grasping the object in a manner appropriate to its use (Carey et al. 1996). Semantic knowledge about what an object is for evidently needs to be provided by the ventral stream for the object to be grasped correctly. This has been nicely illustrated in an experiment by Creem and Proffitt (2001), which showed that healthy subjects too can make ‘functional’ without ‘metric’ visuomotor errors, under conditions of cognitive overload from a concurrent verbal memory task (Creem and Proffitt 2001). Given the close association of apraxia with left-hemisphere lesions, it may be significant that a concurrent visuospatial task did not interfere with grip selection in this experiment (Creem and Proffitt 2001). These observations illustrate the obvious point that one’s acquired knowledge of a manufactured object’s function permits the brain to anticipate what likely use will be made of the object by a person grasping it.

The intimate collaboration between the visual streams when an observer is faced with tools is revealed by fMRI studies even when no act of grasping can take place (since the tools are presented as pictures). As Chao et al. (1999) and Chao and Martin (2000) showed some years ago, viewing tools selectively activates areas in both the ventral stream and the dorsal stream, chiefly in the left hemisphere in both cases (see Lewis 2006 for review). More recently, fMRI studies using functional connectivity analysis have shown that the two areas concerned (in left posterior middle temporal cortex and intraparietal sulcus, respectively) are mutually interconnected (Bracci et al. 2012; Hutchison et al. 2014), in agreement with an earlier DTI study by Ramayya et al. (2010). Evidence consistent with a ventral-to-dorsal direction of transmission comes from a study by Almeida et al. (2013), who have recently shown that increased neural responses to tool stimuli are still observed in the inferior parietal lobule even when the stimuli are transmitted visually only to the ventral stream. (The experimenters achieved this by presenting the tools as chromatically defined red/green isoluminant stimuli, thereby restricting inputs to parvocellular retinal channels).

Interestingly however, Mahon et al. (2007), using repetition MR suppression, have shown that responses to tools in *both* visual streams within the left hemisphere are coded according to action properties associated with the stimuli—not only the tool-responsive areas in the dorsal stream, where this might be more expected. This suggests a complementary dorsal-to-ventral interaction: that is, two-way traffic within a complex temporo-parietal “tool network”. Almeida et al. (2010) have presented supporting evidence for this using continuous flash suppression to one eye, a technique that effectively blocks ventral-stream processing of stimuli presented to the other eye, while allowing dorsal stream processing to proceed (cf. Fang and He 2005). They still found semantic priming effects from such “unseen” stimuli on the naming and categorization of pictures of tools (though not animals). They argue that information about tools extracted from the prime by the dorsal stream (e.g. “graspability”) can be transmitted to ventral stream processing to aid tool identification. Consistent with such dorsal-to-ventral recursive traffic, Gallivan et al. (2013) have found using fMRI and pattern classification methods that information about planned actions is coded to some degree in ventral-stream areas, including the tool-related area.

As an aside, it should be noted here that there is accumulating evidence that semantic knowledge can influence not just the selection of alternative actions, but even the parameters of the movements themselves. For example the known size of familiar objects such as different brands of matchboxes (McIntosh and Lashley 2008), and the use of meaningful as opposed to meaningless objects (Borchers and Himmelbach 2012) have been found to affect grip aperture during grasping. These effects may be attributable to the prior acquisition of visuomotor habits following repeated actions with the familiar objects in the past, rather than to any mis-scaling on the basis of current visual size processing, on the part of the healthy subjects used in the studies. But either way, any full understanding of everyday visuomotor acts must recognize these phenomena and allow that inter-stream communication is probably involved at some stage in their genesis.

### Section summary: what has the ventral stream ever done for the dorsal stream?

The study of visual form agnosia has flagged up a number of visuomotor tasks that the dorsal stream can only perform with the help of crosstalk from the ventral stream. For example, patient D.F. can use simple visual information about shape, width, and orientation to guide her reaching and grasping as accurately as a healthy person, but when presented with more challenging tasks requiring more complex visual analysis her performance deteriorates markedly. We may assume that the brain’s visuomotor control systems rely on ventral-stream mediation to perform these various kinds of supplementary visual analysis. Likewise when a delay is interposed between a stimulus presentation and a reaching, grasping or saccadic response towards it, again D.F.’s performance deteriorates (as discussed earlier). We must infer here again that the ventral stream is required for us to perform this task; the dorsal stream appears to have no ‘memory’ of the stimulus that was presented, and depends on crosstalk from ventral areas. Similarly when asked to report manually a shape she is shown, or to ‘pantomime’ its size or orientation, D.F. is unable to do so: these capacities evidently depend on the mediation of ventral-stream processing. Use of pictorial depth cues in guiding grasping in depth also seems to rely on ventral stream inputs, particularly when binocular cues need to be supplemented or are absent; and the planning of how to grasp an object to optimize end state comfort likewise requires input from the ventral stream.

## Evidence for dorsal-to-ventral traffic

### Shape and orientation discrimination

Although D.F. normally has severe difficulties in distinguishing among rectangular blocks of different aspect ratio, there have occasionally been instances where she performed somewhat better than would have been predicted. In one such experiment, a square and a rectangular block that she could not discriminate between verbally were presented together, and D.F. was asked to pick up one of them (e.g. the square) over a series of trials (Murphy et al. 1996). Although she achieved above-chance success in this task, closer examination revealed that rather than always reaching for the target object directly, as healthy subjects do, she often changed course mid-flight. It was surmised that she was able to monitor the aperture of her grasp as she reached towards one of the objects, and was then able to use this information either to continue her reach trajectory or to change it when she detected that her reach was directed at the wrong object.

D.F.’s ability to use self-cueing extends to the dimension of orientation. Dijkerman and Milner (1997) asked D.F. to copy a single line presented at one of a variety of orientations on a sheet of paper by drawing on an adjacent sheet. According to her performance with ‘slot-posting’ she should not have been able to do this task: but in practice she performed well. The authors observed that D.F. proceeded by first ‘tracing’ a line in the air above the line presented, and then making the same movement on paper with the pencil. But even when D.F. was required to stop tracing in the air, she continued to copy lines far better than chance. To achieve this she would look at the original line for a few seconds on each trial, with her pencil on the other piece of paper, before then quickly drawing her line. Afterwards she explained that instead of explicitly tracing in the air over the line, she *imagined* doing that, while keeping her pencil ready. She then drew her line quickly, before the imagined movement had faded from her mind. When prevented from doing this by having to copy the line as soon as it was presented, D.F. now drew randomly oriented lines bearing no systematic relationship to the line she was shown.

The above findings reflect forms of self-cueing that may not require direct neuronal cross connections. In a later study, however, we found evidence that this self-cueing could be completely internalized: the very act of picking up rectangular blocks raised D.F.’s ability to discriminate the form of the target object from chance to above-chance performance (Schenk and Milner 2006). The authors used a square and an oblong block equated for surface area like Murphy et al. (1996), presenting them one at once. They found that D.F. could name the object while concurrently grasping it at a level significantly higher than when she made judgements without grasping, which remained at chance. The results of control experiments ruled out proprioceptive and efferent cues, supporting the idea that internal cues derived from visuomotor processing could directly influence discriminative responses in D.F. A further test showed that the grasping-induced discrimination improvement disappeared when the target objects differed only with respect to their shape but not their width, suggesting that shape information per se did not underlie D.F.’s grasping in the task. While the results do not mean that D.F.’s conscious perception of the block’s geometry improved during concurrent grasping, it remains a possibility that dorsal-to-ventral signals might have biased her binary decisions to above-chance levels via spared temporal lobe systems.

### Stereoscopic depth perception

In the Dijkerman et al. (1996) study discussed earlier, D.F. was able to perform well at adjusting her handgrip orientation to match the slant of an object, though monocular viewing reduced her performance. In contrast, her perception of slant, as indicated by her ability to match it using a hand-held object of the same dimensions, was poor, falling to chance under monocular viewing. This difference provides another example of the dissociation between perception and action that characterizes D.F.’s visual life. However, the question still arises as to how binocular viewing rendered her able to match object slant at an above-chance level. Given that there are dedicated mechanisms for computing orientation in depth in dorsal stream area AIP (Sakata et al. 1995), might it be that when binocular cues are available to her, D.F. can derive cross-stream benefit from those AIP neurons to inform her slant judgements? D.F. does have a surviving ability to judge depth as tested with Julesz stereograms (Milner et al. 1991; Read et al. 2010), and actually falls within the range of healthy controls when judging slant created with full-field stereograms (Read et al. 2010).^1^ Although D.F. is unable to identify the *shapes* that she can see emerging in stereoscopic presentations, presumably due to her damage to area LOC, she does seem to have distinct percepts of an object located in depth. It is possible that these experiences of depth might be informed by dorsal-to ventral crosstalk.

In a remarkable series of related studies, the stereoscopic perception of curvature has been investigated in nonhuman primates. Srivastava et al. (2009) reported robust selectivity for disparity-defined curved surfaces as well as slanted ones in a high proportion of AIP neurons sampled in the monkey. They noted that this representation of 3D shape features in dorsal stream neurons would provide just the kinds of object parameters needed for programming grasping movements. However, Verhoef et al. (2015) have recently provided evidence that the activity of these curvature-selective neurons in AIP is also related to the monkey’s choice behaviour in a discrimination task between disparity-defined 3-D shapes. The same group had earlier shown that the activity of neurons in part of the anterior inferior temporal cortex (ITC) correlates with trial-by-trial judgements made by monkeys during 3-D shape categorization (Verhoef et al. 2010), and that micro-stimulation of these neurons strongly modulates those same judgements (Verhoef et al. 2012). In their most recent paper on this topic, the same researchers have demonstrated that there are clear causal links underlying these phenomena, with dorsal stream activity playing a determining role in both ventral stream activity and curvature discrimination judgements (Van Dromme et al. 2016). They report that reversible inactivation of the caudal intraparietal area (CIP) reduced fMRI activations elicited by curved surfaces in both AIP and ITC, and also caused a deficit in discrimination. These results provide the first clear causal evidence for the flow of visual 3D information from the dorsal stream to the ventral stream, and identify CIP as a key area for depth-structure processing. The results of this processing appear to be passed on to AIP to inform motor acts, or to the ventral stream to inform perceptual decisions, as and when the current task demands it.

### Section summary: what has the dorsal stream ever done for the ventral stream?

As indicated earlier, these influences of dorsally processed visual features such as width, 2D orientation and figural depth upon the operations of the ventral stream were not predicted by the two-visuals-streams model as outlined almost a quarter-century ago by Goodale and Milner (1992; Milner and Goodale 1993). However, judging from the evidence gathered thus far, it should be noted that the dorsal-to-ventral traffic seems to carry somewhat primitive visual information, based on simple object features rather than anything of a more configural nature. It is the reverse traffic, from ventral to dorsal stream, that seems to carry visual and semantic complexity, thereby allowing us to bring meaning to our actions. This makes good sense within the framework of the Milner/Goodale model.

Indeed, notwithstanding the risks in making inferences based on the less-than-clean lesions in patient D.F., I would suggest that the processing of visual inputs in the dorsal stream appears to be restricted to relatively simple features rather than complex configurations. Support for this conclusion comes not only from neuropsychology, but also from a study using continuous flash suppression in healthy human subjects (Almeida et al. 2010). To quote those authors:

Our results indicate that the dorsal stream, in isolation from the ventral stream, is agnostic as to the identity of the objects that it processes. We suggest that structures within the dorsal visual processing stream compute motor-relevant information (e.g. graspability), which influences the identification of manipulable objects, and is not either about the function of the object or function-specific.

Contrary to this conclusion, a case has been made recently that independent computation of complex shape proceeds in parallel in both visual streams (Freud et al. 2015, 2016). Their argument is based on the perception of images depicting possible and impossible objects in healthy and agnosic subjects. The two patients who were tested using fMRI were impaired at distinguishing possible from impossible objects, and evinced a lower differential activation in their damaged ventral streams, yet the two classes of objects still showed differential activations in the parietal cortex. While it is not impossible that this kind of complex spatial processing occurs independently in the dorsal stream, a perhaps more plausible interpretation would be that a signal is generated in the ventral stream (the right LOC was still differentially responsive in the two patients) that then informs the dorsal stream as to the depicted object’s graspability.

## Concluding thoughts

The approach I have taken to the question of cross-stream interaction is perhaps a biased and idiosyncratic one, emphasizing as it does the value of neuropsychological evidence as a starting point. Excellent reviews in which a more balanced approach has been taken are those of van Polanen and Davare (2015) and Cloutman (2013), the latter of which compares possible similarities between different sensory modalities. What I hope that the present rather selective review offers is a corrective to the surprisingly common view that the original two-visual-systems model of Milner and Goodale postulated two *independent non-interactive* streams of processing. This is particularly ironic given that most of Milner and Goodale’s published research with patient D.F. over the past 25 years, which has provided the backbone of the subsequent development and refinement of the model, specifically documents the *results* *of depleted inter-stream communication*. The failures of her visuomotor ability under various experimental circumstances have consistently been explained by the authors as precisely the result of a loss of inputs to the dorsal stream from the ventral stream.

A model of the two visual streams as fully encapsulated has always been explicitly recognized as untenable by the model’s proponents. Indeed at a general level, a moment’s thought will reveal that the fact of different brain modules doing different jobs and processing information in different ways could never exclude the possibility (even likelihood) that those modules are interconnected and to varying degrees interdependent. Examples disproving such a naïve supposition abound in neuroscience. For example, it has long been known that different sensory modalities interact in the brain to some degree—yet nobody would claim that they should, therefore, be regarded as somehow part of a single system.

## Footnotes

1 D.F.’s stereo perception is however by no means fully normal. The control subjects all showed substantial significant improvements in a condition where the slant was represented only in a strip set between areas of zero disparity (no slant), whereas DF did not. This may result from D.F.’s having lost the scene-based processing mode that seems to characterize ventral-stream processing (Goodale et al. 2004)
